# Perceived Health among Adolescent and Young Adult Survivors of Childhood Cancer

**DOI:** 10.3390/curroncol28010080

**Published:** 2021-02-07

**Authors:** Morgan Young-Speirs, Caitlin Forbes, Michaela Patton, K. Brooke Russell, Mehak Stokoe, Kathleen Reynolds, Fiona Schulte

**Affiliations:** 1 Cumming School of Medicine, University of Calgary, Calgary, AB T2N 4N1, Canada; morgan.youngspeirs@ucalgary.ca; 2 Department of Oncology, Division of Psychosocial Oncology, Cumming School of Medicine, University of Calgary, Calgary, AB T2N 4N1, Canada; caitlin.forbes@ahs.ca (C.F.); sandhm@ucalgary.ca (M.S.); 3 Hematology, Oncology and Transplant Program, Alberta Children’s Hospital, Calgary, AB T3B6A8, Canada; kareynol@ucalgary.ca; 4 Department of Psychology, University of Calgary, Calgary, AB T2N 4N1, Canada; michaela.patton@ucalgary.ca (M.P.); kbrussel@ucalgary.ca (K.B.R.); 5 Department of Family Medicine, Cumming School of Medicine, University of Calgary, Calgary, AB T2N 4N1, Canada

**Keywords:** pediatric, adolescent, young adult, oncology, survivorship, quality of life

## Abstract

Survivors of childhood cancer (SCCs) are at increased risk of late effects, which are cancer- and treatment-related side-effects that are experienced months to years post-treatment and encapsulate a range of physical, cognitive and emotional problems including secondary malignancies. Perceived health can serve as an indicator of overall health. This study aims to (1) understand how a patient reported outcome (PRO) of perceived health of SCCs compares to controls who have not had a cancer diagnosis and (2) examine the relationships between perceived health and demographic and clinical variables, and health behavior. A total of 209 SCCs (*n* = 113 (54.10%) males; median age at diagnosis = 6.50 years; median time off treatment = 11.10 years; mean age at study = 19.00 years) were included. SCCs completed annual assessments as part of Long-Term Survivor Clinic appointments, including a question on perceived health answered on a five-point Likert scale. Data were collected retrospectively from medical charts. Perceived health of SCCs was compared to a control group (*n* = 836) using data from the 2014 Canadian Community Health Survey. Most SCCs (67%) reported excellent or very good health. The mean perceived health of SCCs (2.15 ± 0.91) was not statistically different from population controls (2.10 ± 0.87). Pain (B = 0.35; *p* < 0.001), physical activity (B = −0.39; *p* = 0.013) and concerns related to health resources (B = 0.59; *p* = 0.002) were significant predictors of perceived health. Factors shown to influence SCCs’ perceived health may inform interventions. Exploration into how SCCs develop their conception of health may be warranted.

## 1. Introduction

Treatment of pediatric cancer is a major medical success story. The mortality rate for pediatric cancer has declined by more than 50% over the last several decades in developing countries, resulting in exponential growth in the number of survivors [1]. Currently there are approximately 500,000 survivors of childhood cancer (SCCs) living in North America [2]. Although these numbers are encouraging, SCCs are at risk of experiencing significant medical (e.g., secondary malignancies) and psychological (e.g., anxiety) late effects [3]. Late effects are cancer and treatment-related side-effects that are experienced months to years post-treatment and encapsulate a range of physical, cognitive, and emotional problems. The prevalence of late effects among SCCs is staggering. Ninety-five percent are diagnosed with a chronic health condition by the age of 45 years; 80.5% are diagnosed with a disabling or life-threatening condition [4]. Many of these late effects have severe consequences that can lead to premature mortality [5]. Further, as young adults and later in life, this population is at risk of experiencing challenges related to educational attainment, employment, social relationships, intimacy and fertility [6,7,8]. Due to the growing population and elevated risk of experiencing late effects, it is increasingly important that the health of SCCs be assessed on an ongoing basis.

Perceived health serves as an indicator of overall health, including both physical and psychological dimensions [9]. Perceived health has been shown to be a predictor of morbidity and mortality [10], physical functioning [11], and utilization of health services [12,13]. In oncology specifically, perceived health has been shown to be a predictor of survival [14,15]. Perceived health is a subjective measure, influenced by one’s social and cultural context. The term “health” is not universally defined, allowing respondents to determine what health means to them. Thus, asking respondents to rate their general health requires respondents to reflect on a diverse range of contributing factors that are not specifically covered by other health indicators [16]. Factors including respondents’ psychological state, medical knowledge, previous and present health experiences and physical symptoms may inform their perceptions of their health [16]. The relationship between perceived health and health outcomes is hypothesized to be mediated by the influence of individual perceptions on health behaviors. The Health Belief Model explains how individuals’ perceptions can influence their health behaviors. For example, if it is recommended that an individual takes a specific action, the perceived susceptibility of acquiring an illness, perceived severity of such illness, perceived benefits and barriers of the advised action, and stimulus promoting the advised action, can contribute to the likelihood that the individual will adhere to the recommendation. With perceived health being a comprehensive measure, it may account for the aforementioned health perceptions, influencing an individual’s behaviour. Further, given that perceived health draws on a diverse range of factors specific to the individual, and which may not be covered by other health measures, it may serve as an important identifying indicator of survivors that require additional support [17].

Previous research suggests that health perceptions predict health behavior. Specifically, health perception has been correlated with adherence to care [18]. Patients experiencing less serious disease conditions, such as a sore throat, showed better adherence to care when they perceived their health to be poor [18]. Comparatively, patients experiencing more serious disease conditions, such as cancer, showed worse adherence to care when they perceived their health to be poor [18]. This aligns with findings by Evangliste and colleagues [19] who found that among lung cancer survivors, perceived poor health status was associated with health risk behaviors, including smoking and alcohol use. Specifically, among SCCs, Kazak and colleagues found that young adult SCCs were found to have less adaptive health beliefs, meaning they were more likely to assess themselves as not being as healthy as other people and as being more vulnerable to future health problems [20]. Further, Gibson and colleagues explored survivors’ perceptions of future health and cancer risk among a large cohort of adult SCCs. This work revealed that 31% of survivors were not concerned about their future health, and concern about future health was only modestly higher than a control sample of siblings [21]. However, both Kazak and colleagues and Gibson and colleagues did not assess overall perceived health but measured specific health beliefs. Assessing overall perceived health rather than specific health beliefs may capture more comprehensive responses that incorporate a greater number of psychosocial and physical concerns. Additionally, while both studies explored clinical factors associated with health perceptions, they did not explore the relationship between health perception and health behaviors. Moreover, while Kazak and colleagues found that young adult SCCs had less adaptive health beliefs, Zebrack and colleagues found that among a sample of 176 adolescent and young adult childhood cancer survivors (age 16–28) the majority of SCCs reported excellent or very good health [22]. However, Zebrack and colleagues only reported perceived health, but did not explore the factors which influenced it. Thus, inconsistencies in reported perceived health of SCCs and the lack of exploration between perceived health and its influencing factors suggest that this relationship warrants further inquiry.

Therefore, this study aimed to strengthen the understanding of patient-reported perceived health among adolescent and young adult SCCs who are currently attending a long-term follow up clinic. Specifically, this study had two objectives: (1) to understand how a patient reported outcome (PRO) of current perceived health of SCCs, calculated by completing a retrospective chart review of SCCs at our institution, compared to controls; and (2) to examine the relationships between perceived health and demographic and clinical variables, as well as health behaviors. Based on previous research, we hypothesized that SCCs would not show significant differences in their perception of perceived health compared to population controls. In addition, we hypothesized that experiencing a greater number of health problems and psychological problems would be related to poorer perceived health. Finally, we hypothesized that engaging in risky health behaviors (i.e., binge drinking, smoking and drug use) would be related to poorer perceived health, whereas physical activity would be related to better perceived health. This study will contribute to our knowledge of the factors that are related to perceived health among a population of adolescent and young adult survivors of childhood cancer.

## 2. Experimental Section

### 2.1. Participants

Long-term Survivors of Childhood Cancer: All participants were enrolled in a Long-Term Survivors Clinic (LTSC). The LTSC aims to promote the health and well-being of SCCs by educating and surveilling for the presence of late effects. All patients treated for childhood cancer who receive chemotherapy and/or radiation therapy at the Alberta Children’s Hospital are transferred to the long-term survivor clinic, regardless of diagnosis or treatment for surveillance of potential late effects. The Alberta Children’s Hospital is one of only two pediatric hospitals in Alberta, and thus represents the majority of survivors treated for cancer in southern Alberta. Patients enrolled in the clinic have been off treatment for at least two years. On average, patients visit the clinic once annually. However, some patients who have more recently completed treatment visit biannually.

Control group: The Canadian Community Health Survey (CCHS) is a national cross-sectional survey of the Canadian population that collects information related to health status, health care utilization and health determinants from Canadians aged 12 and over who live in private dwellings in all ten provinces and three territories. The CCHS is a joint effort of Health Canada, the Public Health Agency of Canada, Statistics Canada, and the Canadian Institute for Health Information (CIHI). An area frame and multistage stratified cluster-sampling procedure was used to survey 63,522 households across Canada [19]. Excluded from the target population were persons living on reserves, full-time members of the Canadian Forces, institutionalized populations, children in foster care and certain remote regions. Such exclusions accounted for less than 3% of the total Canadian population. CCHS data is available for public use. Data from the 2014 CCHS cycle was used. For the purposes of this study, we only selected respondents to the CCHS from Alberta to ensure geographical consistency with the participants from the LTSC. In addition, respondents to the CCHS with a previous history of cancer were excluded. Further, given some of the unique characteristics of our sample (i.e., younger cohort), the comparison sample was matched to our sample of survivors by sex and age at a ratio of 1:4, where possible. After matching to a case, the controls were censored so that they could not be assigned to another case. A total of 836 controls were identified.

### 2.2. Procedure

As part of their routine clinic visit, SCCs are asked to complete a patient reported outcome: the Long-Term Survivor Questionnaire (LTSQ), which is mailed to SCCs prior to their clinic visit. The LTSQ asks about current health status. SCCs who do not complete the questionnaire prior to their appointment are asked to complete the questionnaire in the waiting room. As a result, 100% of survivors attending the LTSC completed the questionnaire. Recommendations from the clinic were that questionnaires for survivors <13 years of age be completed by parent-proxy whereas questionnaires for survivors ≥13 years of age be self-reported due to the nature of some of the health questions designed for this age range (e.g., sexual health). Consistent with PROs, responses were reviewed as part of the clinic visit. Following ethics approval, data from the LTSQ from August 2015 to December 2016 were retrospectively collected to capture one clinic visit among the cohort of survivors. 

### 2.3. Measures

Long-Term Survivor Questionnaire (LTSQ). The LTSC is a PRO that was designed to facilitate communication between the LTSC team, the survivor, and their family. This questionnaire is not standardized and has not been validated. Within the LTSQ, perceived health is assessed using the following question: “In general, your health is: (a) excellent; (b) very good; (d) good; (c) fair; (e) poor.” This method has been previously used to assess the perceived health of other populations [11]. The questionnaire also asks about SCCs’ current experiences of health problems using the following question: “Since your last clinic visit have you had any of the conditions below”. Response options for each condition are “yes” or “no”. The survey also asks a series of “yes” or “no” questions regarding health behaviors (e.g., physically active, binge drink, smoke or use street drugs) and health concerns (e.g., fear of cancer returning, fear of experiencing other health conditions, problems finding a doctor, concern about paying for medication).

SCCs were further asked about the following symptoms of clinical depression consistent with the Diagnostic and Statistical Manual of Mental Disorders-5 criteria: “Have you had any of these problems during the last month: (a) loss of interest in daily activities; (b) disturbed sleep patterns; (c) changes in appetite or weight; (d) feelings of sadness, hopelessness, despair; (e) crying easily for no reason”.

SCCs’ Demographic and Clinical Information. Demographic information (i.e., sex and age) and clinical information (i.e., diagnosis, age at diagnosis, treatment, time since treatment, and relapse) were obtained from SCCs’ charts.

The Canadian Community Health Survey. Within the CCHS, perceived health is assessed using the following question: “In general your health is: (a) excellent; (b) very good; (d) good; (c) fair; (e) poor.” Perceived health is assessed using the same question in both the LTSQ and the CCHS. In addition to perceived health, data related to sex and age was retrieved from the 2014 CCHS cycle.

### 2.4. Statistical Analysis

Analyses were conducted in SPSS version 25. To address objective 1, the percentage of SCCs who reported excellent, very good, good, fair or poor health at their 2015/2016 clinic visit was calculated. Further addressing objective 1, a linear mixed model analysis was used to compare the mean perceived health of SCCs in 2015/2016 to the mean perceived health of controls who had not had a diagnosis of cancer accounting for matched pairs. 

To address objective 2, three separate variables were generated to combine similarly reported health problems: pain, depression and health resource concerns. The pain variable encompassed the experience of any pain type, defined as reporting headache, chest, back and/or other chronic pain. The depression variable encompassed the experience of depressive symptoms, defined as reporting loss of interest in daily activities, disturbed sleep patterns, changes in appetite or weight, feelings of sadness, hopelessness, despair and/or crying easily for no reason. The health resource concerns variable encompassed the experience of concerns related to healthcare resources, defined as reporting concerns regarding paying for medications and/or finding a doctor. The numerical values of the new variables were determined by adding the “yes” and “no” responses of all questions pertaining to each new variable for each participant. Thus, taking the pain variable as an example, this variable ranged from 0 to 4, with 0 being no pain and 4 being the endorsement of all 4 painful health conditions (i.e., headache, back, chest and other chronic pain). Subsequently, the strength and direction of associations between the perceived health of SCCs and demographic and clinical variables, as well as health behaviors, were assessed by calculating Spearman’s bivariate rank-order correlations. Results from correlation analyses were used to determine predictor variables for regression analyses to understand the effect of these variables on perceived health.

## 3. Results

### 3.1. Characteristics of the Sample

A total of 209 SCCs (54.10% males) were included in the analyses (Table 1). Survivors were compared to population controls (*n* = 836) matched on sex and age distribution. 

### 3.2. Objective 1: SCCs Perceived Health and Comparison to Control Group

The majority of SCCs reported either excellent (25.36%) or very good health (41.63%), followed by good (26.32%), fair (5.26%) and poor health (1.44%) (Table 2). SCCs reported a mean perceived health of 2.15 ± 0.91, corresponding to ‘very good’, which was not statistically different from the mean perceived health reported by controls (2.09 ± 0.87) (*p* = 0.49). When comparing perceived health of different age groups, linear mixed model analyses accounting for matched pairs revealed that there was no difference between reports from SCC and controls (Table 3). SCCs with excellent perceived health reported, on average, the fewest number of health problems, with a mean of 0.96 ± 1.13 health problems of a possible 20. Comparatively, SCCs with fair and poor perceived health reported 6.75 ± 3.88 and 12.00 ± 1.41 health problems, respectively (Table 2).

### 3.3. Objective 2: Relationships between Perceived Health and Demographic and Clinical Variables, as Well as Health Behaviors

Bivariate correlation coefficients between health and demographic and clinical variables, as well as health behaviors, can be found in Table 4. Linear regression was subsequently used to assess whether clinical characteristics, health problems and health behaviours were related to SCCs’ perceived health (Table 5). Variables entered into the model were selected based on bivariate correlations as well as theoretical assumptions and included: sex, age at diagnosis, time off treatment, total pain, depression, health resource concerns, physical activity. The model was statistically significant, F(7,165) = 9.29, *p* < 0.001, and accounted for 28.3% of variation within SCCs’ perceived health with an adjusted R^2^ = 0.25. Pain (*β* = 0.319; 95% CI = 0.181 to 0.458; *p* < 0.001), physical activity (*β* = −0.371; 95% CI = −0.689 to −0.054; *p* = 0.022) and health resource concerns (*β* = 0.506; 95% CI = 0.117 to 0.895; *p* = 0.011) predicted SCCS perceived health. Thus, for every additional experience of pain, perceived health decreased by 0.319 (excellent health = 1; poor health = 5). Sex, age at diagnosis, time off treatment and depression were not a statistically significant predictors of SCCs’ perceived health.

## 4. Discussion

This study aimed to understand patient reported perceived health of SCCs who are currently attending a long-term follow up clinic. More specifically, the objectives of this study were to understand how current perceived health of SCCs compares to controls and to examine the relationships between perceived health and demographic and clinical variables, as well as health behaviors. The majority of SCCs (67%) reported their health as excellent or very good and was not significantly different from population controls. These findings parallel previous research that has found the majority of SCCs report excellent or very good health and have only modestly higher concerns about future health compared to a control sample of siblings [21,22]. The current study expands this previous work, however, by focusing on current health status among a cohort of survivors who are attending a long term follow up clinic, as well as by assessing the relationships between perceived health and demographic and clinical variables. 

There are several hypotheses that may provide insight into understanding the factors that contribute to similar perceived health among SCCs and controls. Of course, the similarity in perceived health between SCCs and controls may reflect similar objective health status. However, this seems unlikely given the extensive literature documenting the increased rates of health difficulties in SCCs [23,24,25]. Alternatively, the similarity observed in perceived health may be due to differences in knowledge about one’s own health. For example, Bashore and colleagues reported that less than 30% of SCCs or their parents were aware of SCCs’ risk of experiencing late effects and therefore this lack knowledge may be reflected in SCCs’ conception of good health [26]. Finally, SCCs may evaluate their health differently than healthy controls. There is no single universal understanding of what constitutes health [16]. Rather, what constitutes health is subjective and shaped by one’s social and environmental context. When evaluating one’s health status, respondents first interpret the meaning of health, and then decide what factors should be accounted for as components of their health. Both previous and present health experiences likely influence the factors that individuals incorporate into their health assessments. Thus, differences in the health histories of SCCs and controls may contribute to different interpretations of what constitutes health among both groups [16]. 

Pain, health resource concerns and physical activity were related to SCCs perceptions of current health. These findings are novel with respect to SCCs, but parallel research conducted with other populations. For example, experiences of pain have been shown to be predictors of perceived health among older adults [27], and adults with chronic pain [28], chronic disease and disability [28,29,30]. Among healthy adolescents, physical activity has been related to better perceived health [31]. These factors in relation to SCCs’ perceived health have important clinical implications and emphasize the need to screen for these factors in follow-up care. Interestingly, depression, sex and age at diagnosis were not significantly related to perceived health. Although perceived health is considered an indicator of overall health including both physical and psychological dimensions, in SCCs perceived health appears to be driven more by physical elements. This warrants further investigation.

There are several limitations to the present study. First, we were unable to verify whether questionnaires were completed as self or proxy-report. However, the clinic recommended that survivors ≥13 years of age self-report due to the nature of some of the health questions designed for this age range (e.g., sexual health). Certainly, whether questionnaires were completed by the survivors themselves or their caregivers may impact our findings. Generally, research has suggested that self-reports of constructs such as quality of life tend to be rated lower than parent-proxy reports [32]. Additionally, survivors ≥13 years of age may have filled out the questionnaire in the presence of a parent, which may which may have contributed to the under-reporting of responses to sensitive questions such as alcohol and drug use. Moreover, being physically active was not defined objectively, but was rather a subjective assessment on behalf of the participant. This may have resulted in varying interpretations of what it means to be physically active. However, the questionnaire further asked SCCs to describe their activities and how often they participate in them. These follow up questions may have helped to provide some consistency between respondents by prompting reflection on the specific physical activities they participate in when determining if they are physically active. In addition, the study sample included a lower proportion of central nervous system tumor (CNS) diagnoses compared to the general pediatric cancer population. Due to the complicated late effects experienced by CNS survivors, they typically receive primary care from their neuro-oncologist, rather than through the LTSC. The long-term complications of survivors of CNS tumors have been well documented and this may have contributed to higher ratings of perceived health among our study sample. Finally, it is important to recognize that associations are insufficient to draw conclusions on causation. For example, it is unknown if increased physical activity resulted in improved perceived health, or if higher perceived health levels motivated increased physical activity among SCCs. As the above limitations are each related to study design, future research should consider mitigating these factors. 

Despite these limitations, this study has important implications for understanding the perceived health among SCCs. First, this study suggests that many SCCs who regularly attend a follow-up clinic report excellent or very good health. Perceived health has been shown to be a good predictor of morbidity and mortality [10], physical functioning [11] and healthcare utilization [12,13] in other populations. While this is good news, findings should be considered in the context of other research that has found that only 35% of survivors recognize that they could be at risk for serious health problems in the form of late effects [33]. This may be especially important to consider as our adolescents and young adults transition to more independent health care and will require further education to understand their risk of late effects and the importance of continuing to attend a long-term follow up clinic. Future research should target SCCs who do not attend a follow-up clinic as their perception of their health may vary. We might expect survivors who have been lost to follow-up to have better perceived health and, therefore, not feel the need to attend a regular follow-up clinic [34]. However, predictors of compliance with follow-up care are unclear. [35] Further research also is needed to understand the basis of health ratings. In our study, pain and access to health resources were related to perceptions of health. Follow-up clinics should consider these factors during follow-up appointments. Ultimately, these factors may serve as targets for possible interventions for those SCCs with lower perceived health.

## Figures and Tables

**Table 1:** Descriptive characteristics of participants.

	Long Term Survivors		CCHS Controls	*p*
Characteristics	*N* (%)	Median (Range)	*N* (%)	
**Sex**				
Male	113 (54.10)		452 (54.10)	1.00
Female	96 (45.90)		384 (45.90)	
**Current Age (years)**		19.00 (12.00–34.00)		
12 to14 years	38 (18.20)		152 (18.20)	1.00
15 to 17 years	45 (21.50)		180 (21.50)	
18 to 19 years	25 (12.00)		100 (12.00)	
20 to 24 years	57 (27.30)		228 (27.30)	
25 to 29 years	32 (15.30)		128 (15.30)	
30 to 34 years	12 (5.70)		48 (5.70)	
**Diagnosis**				
ALL/AML	82 (39.20)			
Solid Tumor	72 (34.40)			
Lymphoma	39 (18.70)			
CNS Tumor	16 (7.70)			
**Age at diagnosis** (years)		6.50 (0.00–20.15)		
Time since treatment (years)		11.11 (2.51–33.51)		
**Treatment**				
Surgery—yes	114 (54.50)			
Chemotherapy—yes	202 (96.70)			
Radiation—yes	80 (38.30)			
Relapse—yes	14 (6.70)			

Note: CCHS = Canadian Community Health Survey; CNS Tumor = Central Nervous System Tumor; ALL = Acute Lymphoblastic Leukemia; AML = Acute Myeloid Leukemia.

**Table 2:** Number of health problems reported for each level of perceived health among long-term survivors of childhood cancer survivors.

Perceived Health	*N* (%)	Health Problems
		Mean ± SD
Excellent	53 (25.36)	0.96 ± 1.13
Very Good	87 (41.63)	1.65 ± 2.41
Good	55 (26.32)	3.36 ± 4.15
Fair	11 (5.26)	6.75 ± 3.88
Poor	3 (1.44)	12.00 ± 1.41
Total	209 (100)	2.30± 3.29

Note: Perceived health ranges from 1 to 5, where excellent health = 1 and poor health = 5. Health problems scores range from 0–20, where 0 is no reported health problems.

**Table 3:** Perceived health of controls and survivors of childhood cancer by age groups.

Age Group	Survivor		Control		
	*N* (%)	Mean Perceived Health ± SD	*N* (%)	Mean Perceived Health ± SD	*p*
12 to14 years	35 (16.67)	1.94 ± 0.91	151 (17.82)	2.03 ± 0.76	0.57
15 to 17 years	45 (21.43)	2.12 ± 0.95	180 (21.10)	2.02 ± 0.83	0.50
18 to 19 years	25 (11.90)	2.04 ± 0.79	100 (11.61)	2.07 ± 0.90	0.88
20 to 24 years	55 (26.67)	2.20 ± 0.87	228 (26.73)	2.17 ± 0.90	0.83
25 to 29 years	30 (14.29)	2.40 ± 0.98	128 (15.00)	2.19 ± 0.92	0.26
30 to 34 years	11 (5.24)	2.18 ± 0.87	48 (5.63)	2.13 ± 0.98	0.86
Total	209 (100)	2.14 ± 0.90	836 (100)	2.10 ± 0.87	0.49

Note: Perceived health ranges from 1 to 5, where excellent health = 1 and poor health = 5.

**Table 4:** Correlation matrix comparing perceived health with clinical variables, health problems and health behaviors of long-term childhood cancer survivors.

Characteristic	Spearman’s Rank Correlation Coefficient	Sig. (2-Tailed)	*N*
Sex	0.144	0.051	184
Diagnosis	−0.045	0.545	184
Age at diagnosis	0.129	0.08	184
Time since treatment	0.044	0.557	184
Surgery	0.003	0.963	184
Chemotherapy	0.062	0.405	184
Radiation	−0.073	0.325	184
Relapse	0.069	0.352	184
Health problems:			
Frequent headaches	0.282 **	0.000	182
Problems seeing	0.111	0.136	183
Problems hearing	0.026	0.73	183
Dizziness or unsteadiness	0.232 **	0.002	182
Problems walking	0.156 *	0.035	183
Shortness of breath	0.134	0.07	183
Dry eyes	0.073	0.328	183
Dry mouth	0.022	0.772	183
Difficulty swallowing	0.127	0.086	183
Skin changes	0.185 *	0.012	183
Frequently tired	0.306 **	0.000	183
Sensitivity to heat or cold	0.094	0.207	183
Chest pains	0.258 **	0.000	183
Rapid or irregular heartbeat	0.14	0.058	183
Frequent constipation	0.152 *	0.04	183
Frequent diarrhea	−0.011	0.882	183
Frequent need to urinate	0.135	0.07	182
Burning or pain with urination	0.032	0.668	184
Back Pain	0.380 **	0.000	183
Any other chronic pain	0.223 **	0.003	181
Depressive symptoms:			
Problems getting along with family members	0.007	0.926	181
Problems getting along with nonfamily	0.039	0.602	180
Loss of interest in daily activities	0.312 **	0.000	181
Disturbed sleep problems	0.286 **	0.000	181
Changes in appetite or weight	0.297 **	0.000	181
Feelings of sadness, hopelessness, despair	0.239 **	0.001	180
Crying easily for no reason	0.237 **	0.001	181
Health concerns:			
Fear of cancer coming back	0.089	0.239	178
Fear of health problems or second cancer	0.145	0.051	180
Concerns about paying for medicines	0.2 **	0.007	181
Problems finding a doctor	0.170 *	0.022	181
Health behaviours			
Physically active	−0.171 *	0.020	184
Drinking to intoxication	0.092	0.227	173
Smoking	−0.088	0.256	168
Drugs	0.068	0.38	170

Note: * Correlation is significant at the 0.05 level (2-tailed). ** Correlation is significant at the 0.01 level (2-tailed).

**Table 5:** Linear regression model for clinical variables, health problems and health behaviors associated with survivors of childhood cancer.

				Dependent Variable: In the Past 4 Weeks Your Health Is…				
				Unstandardized Coefficient		Standardized Coefficient	95% Confidence Interval for B	
	R^2^	df	F	B	Standard Error	Beta	Lower Bound	Upper Bound
**Model:**	0.283	7	9.285	1.951	0.255		1.448	2.455
Female vs. Male				0.031	0.116	0.018	−0.197	0.259
Age at diagnosis				0.008	0.012	0.053	−0.015	0.032
Time off treatment				0.013	0.013	0.081	−0.011	0.038
**Total pain**				**0.319**	**0.07**	**0.343**	**0.181**	**0.458**
Depression				0.055	0.049	0.088	−0.043	0.152
**Health resource concerns**				**0.506**	**0.197**	**0.184**	**0.117**	**0.895**
**Physical activity**				**−0.371**	**0.161**	**−0.157**	**−0.689**	**−0.054**

Note: Bolded rows indicate statistical significance (*p* < 0.05). Total pain includes headaches, chest pain, back pain and other chronic pain. Depression includes problems with getting along with family, problems with getting along with nonfamily, loss of interest in daily activities, disturbed sleep patterns, changes in appetite or weight, feelings of sadness, hopelessness, despair and crying for no reason. Healthcare resource concerns include concerns related to finding a doctor and paying for medication.

## Data Availability

The data presented in this study are available on request from the corresponding author. The data are not publicly available due to privacy or ethical reasons.
